# Detecting, Tracking and Counting People Getting On/Off a Metropolitan Train Using a Standard Video Camera

**DOI:** 10.3390/s20216251

**Published:** 2020-11-02

**Authors:** Sergio A. Velastin, Rodrigo Fernández, Jorge E. Espinosa, Alessandro Bay

**Affiliations:** 1 Zebra Technologies Corp., London WC2H 8TJ, UK; alessandro.bay@zebra.com; 2 School of Electronic Engineering and Computer Science, Queen Mary University of London, London E1 4NS, UK; 3 Department of Computer Science and Engineering, Universidad Carlos III de Madrid, 28911 Leganés, Madrid, Spain; 4 Facultad de Ingeniería y Ciencias Aplicadas, Universidad de los Andes, Mons. Alvaro del Portillo 12.455, Santiago 7620001, Chile; rfa@miuandes.cl; 5 Politécnico Colombiano Jaime Isaza Cadavid, Carrera 48 No. 7-151 El Poblado, Medellín 050022, Antioquia, Colombia; jeespinosa@elpoli.edu.co

**Keywords:** camera sensor, people detection, multi-object tracking, people counting, deep learning, people tracking

## Abstract

The main source of delays in public transport systems (buses, trams, metros, railways) takes place in their stations. For example, a public transport vehicle can travel at 60 km per hour between stations, but its commercial speed (average en-route speed, including any intermediate delay) does not reach more than half of that value. Therefore, the problem that public transport operators must solve is how to reduce the delay in stations. From the perspective of transport engineering, there are several ways to approach this issue, from the design of infrastructure and vehicles to passenger traffic management. The tools normally available to traffic engineers are analytical models, microscopic traffic simulation, and, ultimately, real-scale laboratory experiments. In any case, the data that are required are number of passengers that get on and off from the vehicles, as well as the number of passengers waiting on platforms. Traditionally, such data has been collected manually by field counts or through videos that are then processed by hand. On the other hand, public transport networks, specially metropolitan railways, have an extensive monitoring infrastructure based on standard video cameras. Traditionally, these are observed manually or with very basic signal processing support, so there is significant scope for improving data capture and for automating the analysis of site usage, safety, and surveillance. This article shows a way of collecting and analyzing the data needed to feed both traffic models and analyze laboratory experimentation, exploiting recent intelligent sensing approaches. The paper presents a new public video dataset gathered using real-scale laboratory recordings. Part of this dataset has been annotated by hand, marking up head locations to provide a ground-truth on which to train and evaluate deep learning detection and tracking algorithms. Tracking outputs are then used to count people getting on and off, achieving a mean accuracy of 92% with less than 0.15% standard deviation on 322 mostly unseen dataset video sequences.

## 1. Introduction

There is a growing worrying tendency, particularly in emerging economies, for people to favor private instead of public means of transport, as reported by Batty et al. [1] and Stead and Pojani [2]. Many governments are actively looking at ways to improve security, safety, and quality of service to encourage public transport patronage, for example, in multimodal forms combined with private transport, in Carrol et al. [3]. An important part of those efforts is to provide transport infrastructure that is both comfortable to users and that provides fast transit times. Railway-based systems (such as metros, trains, trams) have the advantage (over buses and other road-based transport means) of having dedicated tracks where they can travel at the highest possible speeds that signaling and network traffic would allow. Nevertheless, what limits their effective throughput are the points at which passengers need to transfer from/to the vehicles. There are physical parameters that determine the limits of people flow at these transfer points, hence the “dwell times”, i.e., the amount of time that a vehicle needs to be stationary to allow people to board/alight. Examples of such factors are door widths, space (horizontal and vertical “gaps”) between train and platform, payment procedures (e.g., cash or cashless), position of handrails inside the train, floor materials, etc. Some of these factors can be altered during transport operations while some need to be defined when vehicles are manufactured and stations designed. What effect these factors have on passenger flows also depend on cultural factors. Similar considerations exist for Bus Rapid Transit systems and conventional buses that compete for road space with private traffic leading to lower speeds, as studied by Saleh and Ziółkowski [4]. Dedicated bus lanes is a popular way to improve bus services, but they involve the need to enforce against misuse by private vehicles, as discussed by Shalaby [5] and Agrawal et al. [6], for which computer vision-based systems and other technologies have been deployed in many countries, but there are still challenges especially in developing countries as exemplified in studies by Nguyen et al. [7] and Malomo [8]. Therefore, to make public transport networks more attractive, transport engineers would like to adjust physical factors (both at design and operational times) to optimize quality of service and revenues. To do this, they need to understand, and ultimately model, passenger behavior in relation to those factors. Although such studies are possible by direct observation in operational conditions, not all possible alternatives can be investigated (e.g., in an existing metro or bus system, doors or gaps cannot be easily modified). It is also difficult to construct realistic computer-based simulation models if the underlying relationships between behavior and physical environment are poorly known. This is where full-size models, using real people, can help for studying their responses to changes in physical configurations. Although some may argue that it is not possible to replicate operational conditions even with full-size models, because human participants might be aware that they are in an experiment under observation, Fernandez et al. [9] have shown that after an initial period of getting used to an experimental set-up, participants behave in similar ways as they would do under normal conditions.

A typical scenario is the design of metro carriages. There is always a trade-off between passenger comfort and service efficiency, particularly during stop times at stations. In an extreme case, it might be more “efficient” not to have any seats inside a train, but this would be unacceptable to most people. In more practical cases, for many modern trains, such as those serving airports, comfort and safety is important for those carrying heavy luggage and also for those with reduced mobility like the disabled or the elderly. Therefore, how easily, quickly and safely may people board/alight from a train/bus is an interesting matter to study, for example, to determine an appropriate door width and other physical characteristics, to minimize dwell times. Such research increasingly uses video-based observation either in operational conditions or, more likely, e.g., due to privacy concerns, using controlled experiments in simulated environments. One limitation with this approach is that researchers have to spend many hours of valuable time reviewing and annotating the video material, e.g., to count the numbers of people boarding/alighting under the different conditions in their experiments, such as door widths, step height, platform width, passenger mobility, gender, and so on.

Most public transport systems in the world have invested significantly on CCTV (Closed Circuit Television) systems to improve security and safety. The same infrastructure can be used to make measurements to improve quality of service, but the large amounts of video data can lead to human overload and reduced effectiveness.

CCTV systems have largely been designed for human observation. In the last two decades, there have been significant improvements on the camera sensors, in terms of image quality and resolution, and on the digital infrastructure to transmit and store video data. Nevertheless, with the possible exception of Automatic Number Plate Recognition (ANPR) for speed control (e.g., see Ziółkowski [10]) and to analyze passenger and freight movements, as discussed by Hadavi et al. [11], the analysis of the video data of CCTV systems in public transport networks has remained largely based on manual analysis, mostly because of the challenges posed by localizing people, their motions, and, ultimately, their behavior in realistic scenarios. More recently, however, there have been major scientific and technological improvements in computer vision, led by intelligent sensors that use deep-learning methods and that can offer a way forward. Therefore, this paper explores whether such methods can achieve reasonable results in the analysis of people boarding and alighting from a public transport vehicle, thus allowing transport researchers to conduct a larger number of experiments in shorter times.

In this work, real-scale laboratory experiments have been undertaken in the Pedestrian Accessibility Movement Environment Laboratory (PAMELA) of University College London, to test layouts of platforms and vehicles at full-size public transport vehicle. The original aspect of this work is the real-scale laboratory experimentation as a research method, which aims to evaluate the responses of passengers to different configurations which can then be implemented in vehicles and stations to improve operations. Experimental variables are based on observations at public transport systems, such as Transantiago (the Transport System of Santiago de Chile), London Underground, and the public literature in the field.

**Figure 1:**
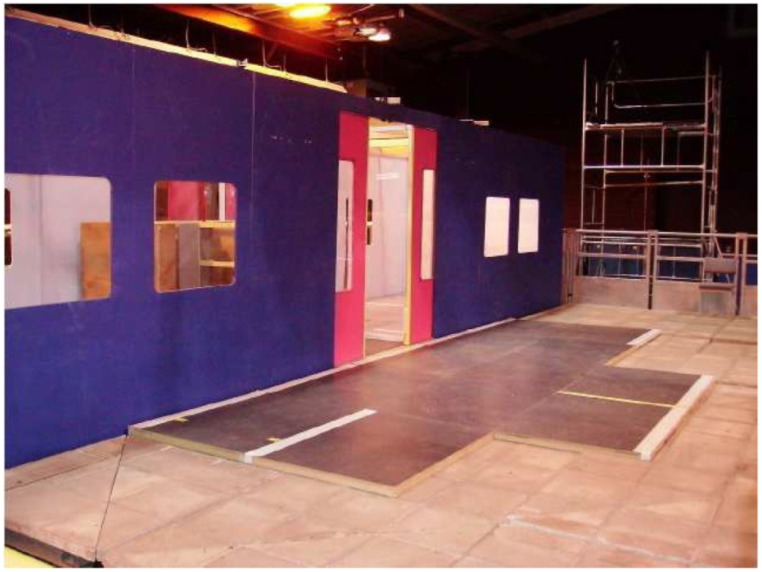
Pedestrian Accessibility Movement Environment Laboratory (PAMELA) configured as a London Underground carriage to study effect of door width and vertical gap [12].

The experiments involved ordinary people getting on and off a real-scale mock-up of a vehicle that were studied to obtain people counts and the times taken to board/alight. Four variables were studied, following earlier work by Fernandez et al. [9]: the fare collection method, the vertical gap, vertical distance between the platform and the vehicle, and the width of the doors and the passenger density inside the vehicle. Figure 1 shows the experimental set-up in which door width and vertical gap was studied.

The main contributions in this paper are:

- A new video dataset of 348 sequences captured by a standard CCTV-type camera is made publicly available. The dataset involves ordinary people boarding/alighting into/from a full-size model of a metropolitan railway carriage under different settings of door widths, step heights and payment method. Counts of people going in/out through the doors are provided for all videos. A small subset of videos has been manually annotated locating and tracking each person’s head, to allow training and testing people detectors.
- With the publication of a dataset, a baseline is established here, as up to now it has not been possible to compare different approaches that use proprietary data. It is hoped that other researchers will now be able to replicate and improve upon these results.
- Three deep learning object detectors (EspiNet, Faster-RCNN, and Yolov3) are evaluated, reaching an F1 close to 90% for the best one. Please see Equation (1) (Section 2) below for a definition of F1.
- Three benchmark trackers Markov Decision Processes (MDP), SORT, and D-SORT) are evaluated, demonstrating a competitive MOTA (Multi Object Tracking Accuracy) of around 80%. Please see Equation (2) (Section 2) below for a definition of MOTA.
- Counts of people are computed for 322 video sequences, obtaining an F1 above 95%

The paper is organized as follows. Section 2 defines the main metrics used in this work. Section 3 highlights some relevant work on image analysis. Section 4 describes the new PAMELA-UANDES dataset (The dataset can be found on http://videodatasets.org/PAMELA-UANDES/). We think that this is a useful and realistic set of data for people working in this area and for which, to the best of our knowledge, there is no clear alternative and that it is hoped will help researchers to identify the state-of-the-art in this field. Section 5 presents baseline results on people detection, tracking and counting obtained using computer vision for this dataset so that future researchers can report improved results. Finally, Section 6 and Section 7 conclude the paper.

## 2. Metrics

Detection performance evaluation is carried out using a standard and well-known set of metrics, such as those proposed by Yin et al. [13] and the VOCchallenge [14]. To determine what are true positive, false positive, and false negative detections (*TP*, *FP*, *FN*), a Jaccard similarity coefficient (also known as Intersection over Union-*IoU*) of 50% is used. So, a true positive occurs when a detection object (*D*) has an *IoU* > 0.5 with a ground truth (*GT*) object, a false positive occurs when a detection does not have an *IoU* > 0.5 with any *GT* object and a false negative occurs when a *GT* object does not have a corresponding detection object. Then,

(1) $$\begin{array}{c} Jaccard=IoU=\frac{D\cap GT}{D\cup GT}\\ precision=P=\frac{TP}{TP+FP}\\ recall=R=\frac{TP}{TP+FN}\\ F1=\frac{2\;P\;R}{P+R} \end{array},$$

where *TP* is the total number of true positives, *FP* the total number of false positives, and *FN* the total number of false negatives. *F*1 is sometimes known as F-score or F-Measure; please see Rennie [15]. Mean average precision (mAP) is the mean of precision over the recall range, and it is also a common metric used for detection and classification problems. All the measures defined in Equation (1) are in the range (0…1).

An almost bewildering large number of tracking metrics have been proposed in the past. Here, the MOT (Multiple Object Tracking) Challenge [16] benchmark definitions are used to obtain different metrics. For simplicity, precision, recall, *F1* and *MOTA* (Multi Object Tracking Accuracy) defined in Bernardin, and Stiefelhagen [17] have been used. In the context of the MOT challenge, precision, recall and F1 refer to object detection metrics, computed as in Equation (1), after tracking and so they test the effect of tracking on object detection. MOTA is a measure that tries to combined various errors that arise in tracking and is defined as,

(2) $$\begin{array}{c} MOTA=1-\frac{\sum_{t}\left(m_{t}+fp_{t}+mme_{t}\right)}{\sum_{t}g_{t}} \end{array},$$

where $m_{t},fp_{t}$ and $mme_{t}$ are the number of misses, false positives, and mismatches, divided by the total number of ground truth annotations for time *t*, respectively. The higher the score the better the results.

## 3. Related Work

### 3.1. People Detection

The computer vision community has been attracted to the problem of people detection for many decades. This is understandable as people’s daily lives are dominated by interactions with other people and therefore there are numerous applications for computer-based people detection, such as Human-Computer Interaction, Surveillance, Safety, Assisted Living, Photography, and, more recently, Autonomous Driving. In the context of the problem addressed here, it is reasonable to think that to measure the number of people getting on/off a Metro and their flows over time, it is necessary fist to detect each individual accurately on each frame (or in regularly time-sampled frames) around the exit/entry area and then to track them in space-time, so that any given individual is only counted once. This has to be done even under crowded or semi-crowded conditions. It can be assumed that the camera sensor is static, although the approach taken here can also work with moving cameras. Early work on pedestrian detection in public places using static cameras, addressed the problem of possible changes in lighting through a process of background estimation and removal by subtraction between the estimated background and the incoming image. The approach of modeling background by a mixture of Gaussians was first proposed by Stauffer and Grimson [18], later improved in Zivkovic [19], and has been extensively used by many authors, e.g., Hu et al. [20]. In its original form, this method will have problems when objects are static or semi-static (as they will be “absorbed” into the background model and when they move they will leave a “ghost” behind). To address this problem, Li et al. [21] propose to model small motions like people turning around or moving their heads. The probability of motion occurrence is predicted from color changes between two consecutive frames, using MID (Mosaic Image Difference) features. Nevertheless, using a background model has significant problems under overcrowding or clutter as mixture models depend on the hypothesis that the background pixels will be observed most of the time, i.e., they are applicable only on light traffic conditions. An alternative approach is to infer people presence through explicit shape analysis. Early work on this include that of Gavrila and Giebel [22] and Mundel et al. [23], but it is difficult to apply in cluttered scenes. The wide variability of human shapes makes explicit modeling very difficult and researchers turned their attention to machine learning (ML) methods. Traditionally, this has required (a) a corpus of data on which to train and test a given ML method, (b) a definition of one or more features extracted from images, and (c) a classifier working in the dimensional space of such features to separate different classes of objects (e.g., people and not people). A seminal and highly cited work addressing these three aspects is that of Dalal et al. [24] that created the INRIA Person dataset containing images mainly taken in street environments and having 614 persons for training and 288 for testing. This work also proposed a feature called Histogram of Oriented Gradients (HOG) that extracts edges and texture features from image regions and that has been shown to be popular for object detection and recognition, e.g., as in Zhu et al. [25], Déniz et al. [26], and Chen et al. [27]. Finally, the features obtained by (HOG) are input to a Support Vector Machine classifier to separate people from non-people. This type of approach (feature extraction, training, classification) became so predominant that it is not possible to review it in the scope of this paper. The interested reader is referred to a comprehensive review by Benenson et al. [28] of traditional pedestrian detection for more details.

However, in 2012 there was a milestone in object recognition with the advent of (a) a very large dataset, ImageNet, of over 1 million examples of 1000 classes made possible by the increasing availability of images on the internet, (b) the availability of very powerful but relatively cheap parallel computing, Graphic Processing Units (GPUs), driven by the games and computer graphics industry, and (c) a revival of neural networks in the form of Convolutional Neural Networks (CNN) that made it practical to deploy neural networks with many “hidden” layers. This became known as “deep learning”, illustrated by the seminal paper of Krizhevsky et al. [29] and has since dominated the field of object localization and recognition. The previous approaches were then called “hand-crafted” and deep learning has become the most popular way to deal with pattern recognition problems, in particular, for object detection and tracking (for an extensive survey; please see Brunetti et al. [30]). The main advantage of deep neural networks is that, provided that there are large amounts of representative annotated data, such systems learn to extract appropriate features (in a serial pipeline of feature extraction of increasing abstraction, e.g., from edges to object outlines, saliency maps, etc.) that are effective to distinguish between different classes on objects. Indeed, the final classification stage becomes less critical.

Recent advances on Deep Learning achieve object detection/classification in an image through one of two main methods, as discussed by Soviany and Ionescu [31]. In the first class, there is a first stage with a Region Proposal Network (RPN) to generate regions of interest. In a second stage those regions are used for bounding box regression and object classification. These detectors are accurate but slow. An alternative family of detectors are the so-called Single Stage (or Single Shot) Detectors (SSDs) that approach object detection as a regression problem, analyzing the input image to learn class probabilities and bounding box coordinates. These models are much faster, even real-time, but can have issues with detecting small objects or with objects that appear too close in the image.

For example, recently, Zhang et al. [32] report a method based on Tiny Yolo, to detect people entering/leaving a bus, training a model on the upper body parts of passengers. As in this work, they had to train with specific data. However, they do not give exact performance metrics, and it is not clear if their dataset is public, preventing verification. No counts are attempted. Guo et al. [33] propose a model called MetroNet to detect people inside a metro carriage, focusing on processing speed with low computational resources. Again, they had to create a dataset (SY-Net) with 1503 images on which to compare MetroNet with Faster-RCNN, SSD, and Yolov3. They use miss rate (MR=1−recall) as the main performance metric, reporting a best MR of around 46%. It is not clear if their dataset is public. A passenger flow counting system for buses is reported by Hsu et al. [34] using an SSD detector and particle filter tracker to then obtain counts. Although the results are good (F1 of around 94% for people detection and almost 92% for counting), their data is proprietary and apparently not publicly available. Liu et al. [35] report a method to measure passenger flows in metros using Yolov3 optimizing its anchors and thus obtaining flow accuracies of 95%. In their case, the camera is mounted on the door frame looking vertically down, which facilitates detection and counting. There is no indication that their data is public.

To present a baseline for the new public dataset presented here, three detectors have been selected: Faster-RCNN, EspiNet, and YOLO (single shot).

#### 3.1.1. Faster R-CNN

Faster R-CNN (proposed by Ren et al. [36]; also see Wang et al. [37]) is a widely used region-based architecture for object detection, that is often used as a benchmark to compare competitive approaches, e.g., as reported for car detection by Benjdira et al. [38]. The method was inspired by earlier work by Girshick et al. [39] and Fast R-CNN [40], combining features extracted from a fully convolutional network and at the same time performing both region proposals and object detection. The model shares the same convolutional layers for the RPN (Region Proposal Network) and the object detection network. The feature map generated by the CNN network is traversed by a sliding window, which generates *k* (9) potential bounding boxes with scores relating to the confidence on the detected object. These potential bounding boxes are related the common aspect ratios of the objects and are called anchor boxes. Bounding boxes and scores are generated by the RPN per position in the image for each anchor box.

#### 3.1.2. EspiNet

EspiNet, proposed by Espinosa et al. [41,42], is a region-based detector model which is inspired on Faster R-CNN. The difference lies in a reduced number of convolutional layers and so a reduced number of parameters to learn (less that 2.3 million) compared to Faster R-CNN and other models.

#### 3.1.3. YOLO

You Only Look Once (YOLO), proposed by Redmon et al. [43], is a single stage detector, with a convolutional network which extracts features, generating a feature map later flattened an analyzed to regress parameters of bounding boxes and classes for the objects detected. More recent versions of YOLO (Yolov3 also by Redmon and Ali [44], Yolov4 by Bochkovskiy et al. [45]) use multi-label classification and logistic regression for objectness score, plus a feature pyramid to deal with varying object sizes. YOLO has become very popular because it can work in real-time and many optimized implementations exist, e.g., for embedded applications.

### 3.2. Multiple Object Tracking

Object tracking in images is about estimating the trajectories of objects in the image plane as they move around a scene; see, for example Yilmaz et al. [46] and Ojha and Sakhare [47]. It involves locating each target in subsequent video frames after it has been localized by a detector. This approach is normally referred to as tracking by detection. It may involve predicting each object’s position in subsequent frames, matching objects between adjacent frames so as to obtain a history or trajectory for each object. Some techniques are used to extract dynamic attributes, including appearance changes, positional speed, direction of movement, and so on. Most tracking algorithms follow one simple principle: objects in two adjacent frames are likely to be the same if a distance measurement between them is small. That distance can be physical separation, appearance, speed, etc. The comparison between currently tracked objects and new detections is sometimes known as data association, i.e., given a known set of objects being tracked at time *t* and a new set of objects detected at time t+1, how to associate the new objects to existing tracks and how to deal with new objects and those that have left the scene. This process needs to take into account both missing and multiple detections for a given object.

Point trackers, silhouette-based tracking, and kernel-based tracking are categories of tracking (see survey by Yilmaz et al. [46]). While point trackers demand detection in every single frame, contour- or kernel-based tracking requires the objects first appearance on scene. For vehicle tracking, deep features are associated combining point tracking and object appearance as, for example, done by Hou et al. [48] with D-SORT to track vehicles.

While single object tracking is already a complex task, Multiple object tracking (MOT) is even more challenging, as it involves localizing and following multiple objects during the video sequence, taking into account occlusion, entry, and exit of objects in the scene.

#### 3.2.1. Kalman Filter Tracking

The Kalman Filter (KF) is a classical and still popular point tracker, because of its simplicity as an on-line recursive process and a well-understood mathematical foundation. A detection process identifies possible dynamic targets represented in its simplest form by a point in 2D or 3D space. Such measurements are assumed to contain Gaussian noise. It is also assumed that objects have an underlying physical model, e.g., a constant acceleration model, represented by state variables. The task of the KF is to provide optimal state variables estimates, given the input measurements and past estimations. For multiple objects, there is a separate data association process that matches detections to predicted state values for the current video frame, typically using some distance measure (Euclidean, Manhattan, etc.). Although simple motion and noise models have been fairly successful when dealing with rigid objects, such as vehicles, they are less successful for pedestrian monitoring, e.g., because of sudden turns or occlusions. Clearly, the filter may suffer when detections are poor. Conversely, some poor detection conditions may be overcome by the filter, e.g., a missing detection or an occlusion can be “smoothed” out by temporarily replacing them with object predictions from past observations.

#### 3.2.2. Appearance-Based Tracking

One of the problems with point-based trackers, such as the KF, is that objects are represented by simple state variables, such as position/speed. As clutter increases and movements become more complicated, it is increasingly difficult to associate point detections with predicted object positions. For non-rigid objects, such as pedestrians, the localization prediction is not always simple, as people can have non-linear motion patterns (e.g., a sudden turn) which are difficult to model and so visual appearance can be a useful cue. This is what is used directly by the Kernelized Correlation Filter (KCF), e.g., Henriques et al. [49], under the assumption that each object has a distinguishable signature. The well known Kanade–Lucas–Tomasi (KLT) tracker is an example of an approach that is based on looking for objects localized in the previous frame(s). This type of tracker only requires objects to be independently localized once and then, ideally, they are detected/followed by appearance alone. This might not be trivial in the presence of occlusions. Bagherpour et al. [50] present an interesting approach combining KLT and KF, but used only on semi-frontal pedestrian images, where people appearance tends to be more discriminative than in the case considered here of near overhead images. Usually color, texture and shape features are used to recognize or re-identify a person (as in Cong et al. [51], Coniglio et al. [52], and Kuo and Nevatia [53]), so that tracking has been able to borrow methods originally devised for image retrieval and person re-identification. For example, Simonnet et al. [54] used DTW (Dynamic Time Warping) to calculate a distance between two observations made at different times and thus re-identify a person. Pedagadi et al. [55] proposed a method based on a local Fisher discriminant analysis to address re-identification that could be used in a tracking context. In KCF, the background is over-sampled to give more context information to the tracker. The idea is explored here as discussed in the experimental portion of this paper, Section 5.

Other tracking methods that have shown promise elsewhere have been disappointing in this case, including the rather slow TLD (Tracking Learning Detection) presented by Kalal et al. [56] and the less accurate Struck proposed by Hare et al. [57] (poor accuracy). A notable exception is MDP (Markov Decision Processes) tracker put forward by Xiang et al. [58], where tracking is modeled as a Markov process that includes reinforcement learning to address the missing detections and data association problems. It should be possible to use deep features within this tracker’s framework. As mentioned earlier, deep learning is the currently most popular approach for object detection and indeed researchers have proposed methods for tracking, including Wojke’s et al.D-SORT [59], based earlier SORT (Simple Online Realtime Tracker) tracker by Bewley et al. [60], where deep features are used for data association. The baselines presented here include the use of these three trackers. There is a significant amount of reported work on multiple object tracking reviewed by Luo et al. [61], including recent surveys on deep learning techniques by Ciaparrone et al. [62], Shuo et al. [63], and Li et al. [64].

### 3.3. Challenges

**Figure 2:**
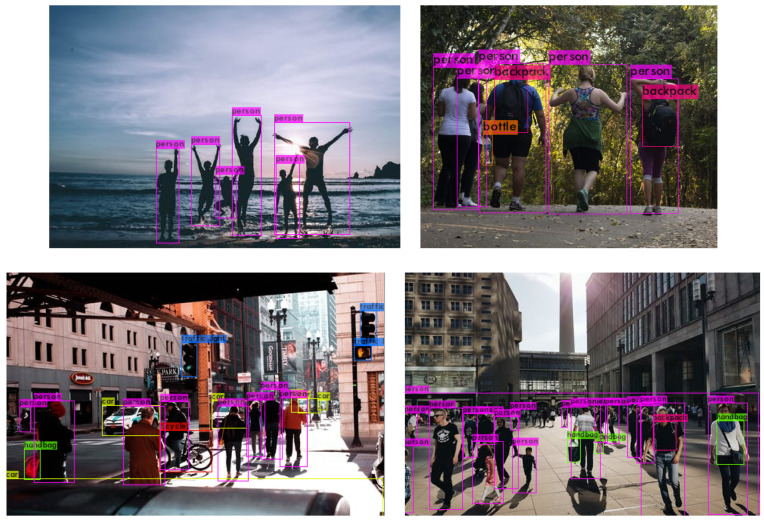
YOLO predictions for images with people, people detections shown in magenta (image source: http://pexels.com).

**Figure 3:**
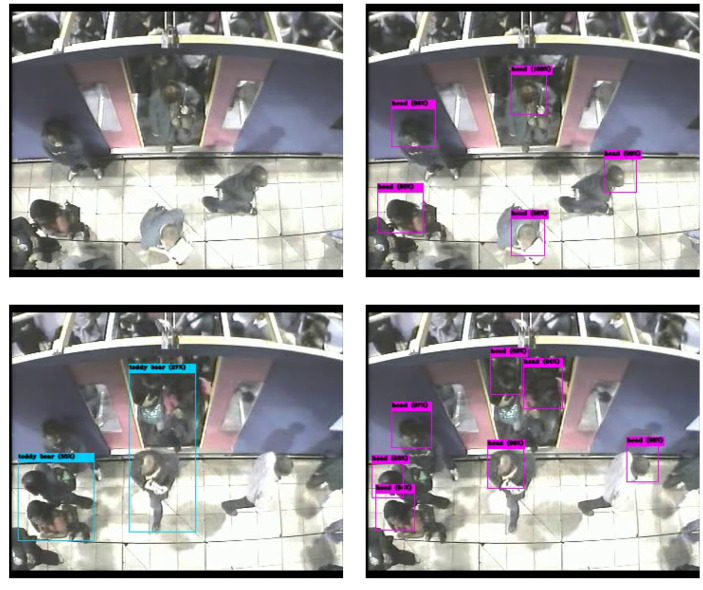
YOLO predictions. (**Left**) pre-trained on COCO; (**Right**) trained on this dataset.

It is clear that deep learning as shown significant success in object detection. There have been many different architecture proposals and variants in the literature, such as CenterNet [65] (Duan et al.), EfficientNet [66] (Tan et al.), RetinaNet [67] (used by Wang et al. for ship detection), Faster-RCNN proposed by Ren et al. [36], SSD discussed by Soviany et al. [31] in comparison with other detectors, Bochkovskiy’s YOLO [45], etc. For object detection, it is popular to test and compare such architectures using the COCOdataset introduced by Lin et al. [68] that contains 80 object classes, including persons. For example, Yolov4 pre-trained on the Coco dataset, obtains impressive results in previously unseen images as shown in Figure 2. However, using the same model to detect people in the new dataset produces poor results, as shown in Figure 3. On the top row (left), the pre-trained model fails to detect any person. On the bottom row (left), the pre-trained model incorrectly outputs two bounding boxes labeled as “Teddy Bear”. The images on the right have been obtained after training a Yolov3 model specifically on this dataset. Although to the human eye the images are still of people, the change in view angle has an adverse effect, whereby many people are not detected. This illustrates that many deep learning methods still have to rely on manual annotation of large amounts of data, even when detecting the same class of objects, but with different views. Many of the popular datasets for people detection, including Coco, mainly contain images of people captured on semi-frontal views standing/walking, limiting their use. Research is still needed to solve this general problem of adapting to different view in the same domain, let alone to different domains. In more general terms, these methods can only capture the appearance of objects for a certain camera view but are still unable to learn underlying properties of objects.

## 4. PAMELA-UANDES Dataset

**Figure 4:**
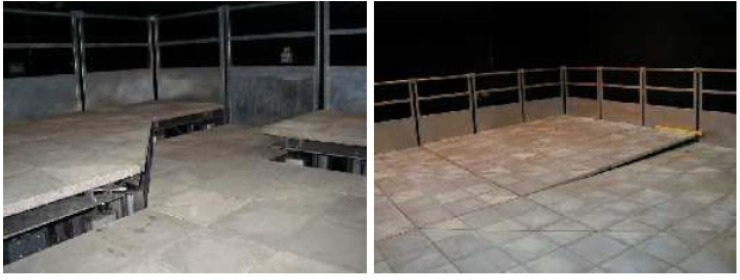
Examples of PAMELA walking areas.

The Pedestrian Accessibility Movement Environment Laboratory (PAMELA) [69] is a multi-sensor facility at University College London (UCL) in the UK, designed for the assessment of pedestrian movement in many application domains (transport, health, architecture, etc.). An interesting characteristic of PAMELA is that it is possible to shape its environment (walls, walking surfaces, lighting, sound) to suit the experiments. The walking area consists of individual 1.2 m^2^ modules making up a total of 80 m^2^. Some examples are shown in Figure 4.

**Figure 5:**
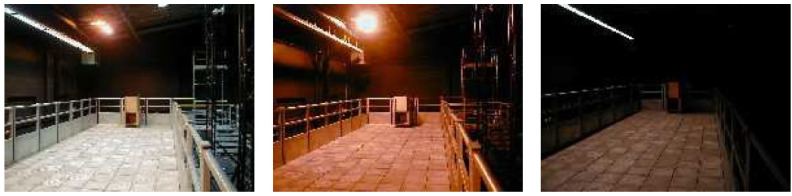
Examples of different lighting conditions in PAMELA.

In terms of lighting, the facility provides control over a wide range of situations from simulation of daylight to moonlight (Figure 5). Video recordings can be made simultaneously from various angles (typically with 8 or more cameras). Note, however, that these cameras are not synchronized nor calibrated.

The video recordings for the dataset used in this work were obtained from a simulated scenario of a London Underground train as first reported by Fernandez et al. [12], covering two door widths (800 and 1600 mm), three step heights (0, 150, and 300 mm), and, in the case of boarding, whether people used a card-based payment system (as per buses) or none (as per trains).

Given the angle of the camera (please see Figure 3), the working hypothesis is that head shapes are discriminating enough, and once people are located they could be tracked from frame to frame to lead to measurements of counts and flows.

Part of the PAMELA-UANDES dataset has been manually annotated, using the ViPERtool [70], so as to investigate computer vision algorithms to automate the process of analyzing such images. Manual annotation is a major task as it took about 2 full person-months to annotate a relatively small subset that consists of 15 video sequences: 8 of people alighting (referred to as “A” videos) and 7 of people boarding (referred to as “B” videos). They last between 1 and 2 min and have a spatial resolution of 352 × 288 at 25 frames per second. The spatial resolution is set by the capturing process that consisted of standard interlaced PAL frames, so 325 × 288 was chosen to avoid interlaced effects and to maintain the width/height ratio.

Videos A_d800mm_R1..4.mpg and B_No_d800mm_R1..4.mpg are used for training while A_d800mm_R5..8.mpg and B_No_d800mm_R5..7.mpg are used for testing. Ground truth files are in ViPER and CSV formats containing, for each pedestrian, a unique identifier, the bounding box around their heads, and the frame numbers in which they appear.

## 5. Experiments

**Figure 6:**
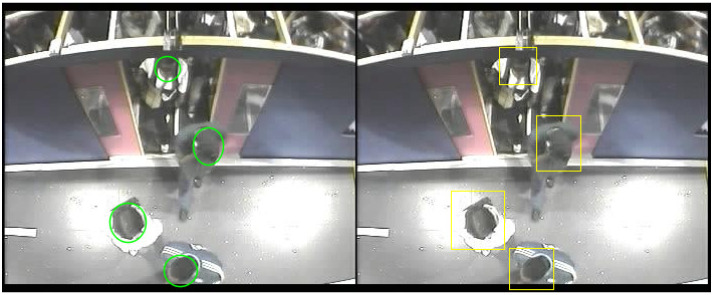
Ground Truth modification. (**Left**) Original ground truth annotation. (**Right**) Expanded bounding boxes.

The PAMELA-UANDES dataset contains a total of 14,834 training images and 13,237 testing images. In both cases, boarding and alighting cases are more or less similar in number. As seen on the left of Figure 6, the original annotation consists of image coordinates of an ellipse defined by an enclosing rectangle ($tl_{x}$,$tl_{y}$, *w*, *h*), where $tl_{x}$, $tl_{y}$ are its top left *x* and *y* co-ordinates, and *w*, *h* are width and height. Following the literature, e.g., Wolf et al. [71] and Dalal et al. [24], it is also hypothesized here that expanding the ground truth bounding box to include part of the background context may help detection. The new bounding box is computed as:

(3) $$\begin{array}{c} \left(c_{x},c_{y}\right)=\left(tl_{x}+\frac{w}{2},tl_{y}+\frac{h}{2}\right)\\ \left(w_{e},h_{e}\right)=\left(w\left(1+f\right),h\left(1+f\right)\right)\\ gt_{e}=\left(tl_{x}-\frac{c_{x}}{2},tl_{y}-\frac{c_{y}}{2},w_{e},h_{e}\right) \end{array},$$

where ($c_{x}$,$c_{y}$) is the (unchanged) object’s centroid, *f* is the expansion factor (in the range 0..1), ($w_{e}$,$h_{e}$) the new (expanded) width and height, and $gt_{e}$ is the expanded object’s bounding box. The effect is illustrated on the right of Figure 6.

The models were trained from scratch, without using any pre-trained model (such as ImageNet) for a fairer comparison. For the same reason, please note that anchors have not been optimized nor data augmentation used. EspiNetV2 took 12 h to train (Windows), Faster R-CNN 18 h (Windows), and Yolov3 14 h (Ubuntu). An Nvidia Titan XP GPU is used for training and testing. Detection performance evaluation is carried out using the metrics defined by Equation (1) (Section 2).

### 5.1. Detection Results

**Figure 7:**
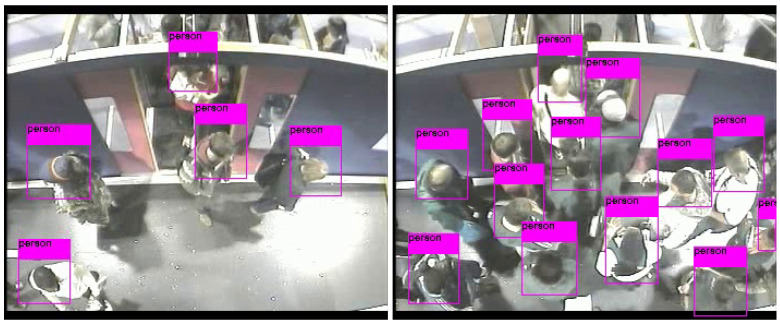
Illustrative detections. (**Left**) Alighting (simpler case); (**Right**) boarding (more complex case).

The detection performances of the three models are evaluated and compared on the 13,238 unseen examples more or less balanced between boarding and alighting examples. The results have been computed using the MOT (Multi Object Tracking) Challenge Development Kit [72]. Results are shown in Table 1 for expansion rates of 0%, 25%, and 50%. Bold figures indicate the best results in each row. The mean and standard deviations are computed over the seven testing sequences. F1 and, to a lesser extent, mean average precision mAP, are good indicators of detection capability. Note that in a real application, F1 is a better indication of performance as it indicates the possible balance between precision (and hence of false positives) and recall (indicative of false negatives), for a given detection confidence threshold. In the cases considered here, Yolov3 is likely to have a precision and recall performance of around 89.5% (when setting an operational point that balances precision and recall). It can be observed that Faster R-CNN and Yolov3 outperform EspinNetV2 in all tests by a margin of around 13–20% in F1. Yolov3 is fairly stable across the different expansion rates and outperforms Faster R-CNN in F1. Faster-RCNN and EspinetV2 show some improvement for a 50% expansion rate (the similar behavior is expected as EspinetV2 is derived from Faster-RCNN). Finally, it should also be noted that Yolov3 has inference (detection) times around five times faster than Faster-RCNN. Figure 7 shows a couple of examples of people detection.

### 5.2. Tracking Results

**Figure 8:**
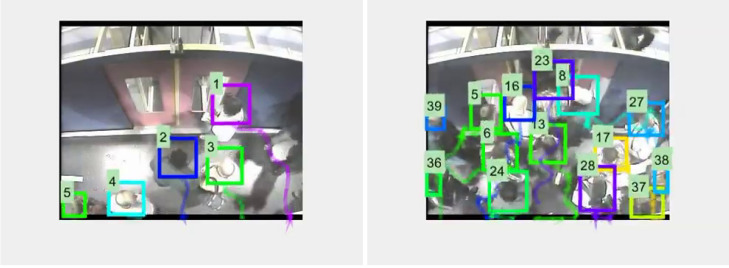
Illustrative tracking example. (**Left**) A simple case; (**Right**) a more complex case. The numbers and colors correspond to unique person identifiers, the trailing tails show their trajectories.

Tracking results from MDP, SORT, and D-SORT trackers have been compared, using the three different detectors and expansion rates. Figure 8 shows a couple of typical tracking examples, while Table 2, Table 3 and Table 4 show tracking metrics obtained with MDP, SORT, and D-SORT, respectively. In each table bold figures indicate the best results for each row. As per the detection experiments, better results are obtained by Faster-RCNN and Yolov3.

In terms of the MOTA metric, best results (81.38) are obtained with SORT/Faster-RCNN/50% followed closely (80.54) by D-SORT/Yolov3/25%. It is reasoned that the angle of capture and the focus on heads, has reduced the amount of occlusions that MDP (via reinforcement learning) and D-SORT (using deep features and cosine distance similarity) have been designed to cope with, and so their particular strengths are not fully stretched. Therefore, the simpler SORT tracker is still able to produce good results with the top two detectors. It is noted that with the poorer detector, none of the trackers do too well (MOTA < 55). It is also noted that the Yolov3/D-SORT combination seems to be less affected by expansion rate; therefore, this combination is used to investigate counting performance, as described in the next sub-section.

### 5.3. People Counting

**Figure 9:**
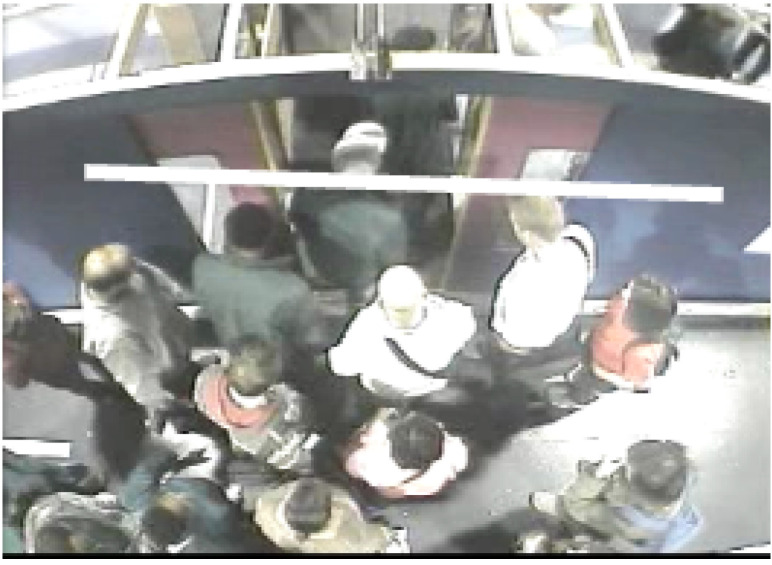
Counting setup: an imaginary line on the image is considered and the tracking results are used to count people crossing the line up (boarding) or down (alighting).

As indicated earlier on, one of the main requirements for transport engineers in this area is to count the number of people entering and exiting the transport vehicle. Once people are detected and then tracked, counting is relatively simpler. In this case, an imaginary line, with a conventional equation x=my+b, across the image is considered as shown in Figure 9. Then, at any given time *t*, for each person, its running average position $\left({\bar{x}}_{t},{\bar{y}}_{t}\right)$ is computed, for all its positions up to time *t*, starting from its first appearance. For example, for a person alighting, this running average would tend to have decreasing values in ${\bar{y}}_{t}$ as *t* increases. When that averages moves from one side of the line to the other (by a given threshold), the person *triggers* a count (down, or alighting, if it originated above the line and up, or boarding, otherwise).

Considering the tracking results (please see Table 4) and taking into account the significant computational advantages of Yolov3, that combination (at an expansion rate of 25%) has been chosen to provide a counting baseline for future researchers. For a given direction of flow (boarding, alighting), true positives/negatives, false positives/negatives are defined as given by Table 5. For example, for boarding, a down-going count would be a false positive, while an undetected person would be a false negative. Note that true negatives are not computed.

Given the definitions in Table 5, in addition to the conventional metrics given in Equation (1) (Section 2), accuracy is defined as follows (note that true negatives are not included):

(4) $$\begin{array}{c} accuracy=\frac{TP}{\left(TP+FP+FN\right)} \end{array}.$$

The evaluation of counting has been done on the videos known as “2008” in the dataset. These consist of 145 alighting sequences (most with 50 people alighting from the carriages) and 177 boarding sequences (most with 27–29 people boarding), under different conditions of crowdiness, depending on physical factors, such as door width. For alighting counts, the imaginary line is y=144 and for boarding y=85. The overall results are summarized in Table 6.

## 6. Discussion

One of the major difficulties in assessing what is the current state-of-the-art in this field has been the lack of representative datasets with number of examples sufficiently large to train modern machine learning methods. Recent works in this area include the use of Tiny YOLO to detect passengers in buses by Zhang et al. [32], a proposal called MetroNet also to monitor passengers in buses described by Guo et al. [33], passenger flow counting by Hsu et al. [34], and metro passenger flows using Yolov3 in Liu et al. [35], which uses proprietary data captured by the authors, so it is not possible to objectively compare results. The absence of data can be explained by the need to observe real people in a natural environment and privacy concerns prevent or limit the publication of such data. What had been proposed is the use of full scale models using people who are habitual public transport users who have given their informed consent to volunteer in these experiments and for the video recordings to be made public. Previous studies show that after a relative short period of getting used to the experimental model, people behave naturally. This has avoided the use of actors, something typical in computer vision-based human behavior studies that have significant biases in terms of age, gender, and levels of fitness. Therefore it is likely that with this dataset and with the baseline results presented in this paper, it will be possible for researchers in this field to establish and improve upon the state-of-the-art.

In terms of the scope of this work on transport engineering, first the method presented showed that annotation time can be reduced between 4 to 5 times, in particular by the ability to track passengers, i.e., where they are located and from where passengers are coming or going once on the platform. The results have an accuracy comparable to human annotators, specially in crowded conditions where manual annotation can lose 1 or 2 people in each boarding/alighting process. Secondly, there is potential for the method to be used in managing the flow of passengers on platforms and inside vehicles, which allows evaluating measures for the control and channeling of passengers. The experiments in the laboratory have shown that the management of passenger flows, such as marking the platform, allows reducing the times of getting on and off by more than 30%. Considering that the dwell time represents approximately 30% of the time in a public transport vehicle, the travel time between stations could be reduced by 10%.

In addition to on-going work on full-size models for passenger monitoring on more complex environments, such as bus stops, further laboratory studies with this technique that are being planned aim to answer the following questions: (a) What is the best combination of vertical and horizontal gaps between the platform and the vehicle to reduce the dwell time? (preliminary studies have shown that the answer is not always zero). To what extent does this combination differ for specific groups of passengers, e.g., elderly people and people with reduced mobility? (b) What is the effect of the density inside vehicles and on platforms on passenger boarding and alighting times and, in turn, on dwell time? (c) What is the influence of the location of the vertical and horizontal handrails, as well as the seating layout, on boarding and alighting times? Can this policy reduce the dwell time? (d) To what extent can the management of passenger on the platforms encourage appropriate behavior to reduce the dwell time? (e) Are there other lines of action, not yet identified, to improve the boarding and alighting processes, safety, and dwell time?

## 7. Conclusions

This work has presented an approach based on machine (deep) learning to locate, track, and count people in a public transport environment, exploiting the full-size PAMELA experimental facility. A new public dataset has been introduced. The main challenge in this dataset is the angled camera view that defeats people detectors trained with popular datasets, but that it corresponds with typical sensor position in this kind of environment. This illustrates the fact that although data-driven learning approaches can produce impressive results, their generalization capabilities are still below that of human observers, leading to the need to capture application-specific data that then has to be manually annotated at significant costs. This is a general observation applicable to many other computer vision application domains.

In terms of sensor processing, the performance of three different state-of-the-art detectors and trackers were evaluated to create a baseline for this dataset. Computational efficiency led to the selection of D-SORT and Yolov3 to assess people counting, one of the main requirements of transport engineers in this domain. Counting accuracies were very satisfactory and provide a good challenge for future researchers to improve upon.

Future work will look at refining the use of spatio-temporal cues provided by video sequences in a detection-by-tracking manner so as to feedback tracking into the detector, rather than the current uni-directional sequential process where a tracker might not recover from detection errors. Additional computational experiments are planned to improve results through optimizing anchor definitions and augmenting data.

## Figures and Tables

**Table 1:** Detection results (all values in percentages, means over all video sequences, ± indicate the standard deviation σ).

%Expansion	Metrics	EspiNetV2	Faster R-CNN	Yolov3
0	Rec	73.0 ± 5.3	**86.9** ± **4.8**	84.2 ± 7.8
	Prec	68.2 ± 10.0	80.7 ± 3.1	**96.2** ± **1.1**
	F1	70.2 ± 6.7	83.6 ± **3.6**	**89.6** ± 5.9
	mAP	74.8 ± 5.9	87.3 ± **3.2**	**91.0** ± 4.1
25	Rec	67.8 ± **4.3**	84.6 ± 4.4	**84.7** ± 7.5
	Prec	73.3 ± 7.7	84.7 ± 3.5	**95.6** ± **1.9**
	F1	70.2 ± 4.3	84.6 ± **3.3**	**89.7** ± 5.1
	mAP	74.8 ± 3.9	87.6 ± **3.0**	**91.3** ± 4.1
50	Rec	69.6 ± 4.9	**88.7** ± **4.7**	84.4 ± 6.9
	Prec	75.9 ± 6.4	88.6 ± 2.4	**95.5** ± **0.6**
	F1	72.4 ± 3.9	88.6 ± **2.9**	**89.5** ± 4.1
	mAP	76.7 ± 3.9	**91.8** ± **2.7**	91.0 ± 3.4

**Table 2:** Tracking results: Markov Decision Processes (MDP) (all values in percentages, means over all video sequences, ± indicate the standard deviation σ).

% Expansion	Metrics	EspiNetV2	Faster R-CNN	Yolov3
0	Rec	79.58 ± 5.64	**89.53** ± 5.04	81.98 ± **4.75**
	Prec	62.79 ± 11.77	**85.71** ± **3.22**	85.65 ± 6.50
	MOTA	28.85 ± 24.65	**74.43** ± **7.29**	67.71 ± 9.38
	F1	69.78 ± 8.39	**87.55** ± **3.74**	83.65 ± 4.51
25	Rec	73.07 ± 5.31	**85.98** ± **4.87**	84.68 ± 5.65
	Prec	68.23 ± 9.59	84.47 ± **3.73**	**89.46** ± 4.31
	MOTA	36.78 ± 15.25	69.86 ± **6.84**	**74.41** ± 7.90
	F1	70.26 ± 6.06	85.14 ± **3.49**	**86.92** ± 4.23
50	Rec	75.61 ± 5.28	**89.20** ± **5.26**	82.94 ± 5.73
	Prec	72.34 ± 7.83	87.70 ± **2.51**	**88.32** ± 6.43
	MOTA	45.36 ± 11.93	**76.49** ± **5.88**	71.47 ± 9.37
	F1	73.71 ± 4.96	**88.37** ± **3.22**	85.39 ± 4.74

**Table 3:** Tracking results: SORT (all values in percentages, means over all video sequences, ± indicate the standard deviation σ).

% Expansion	Metrics	EspiNetV2	Faster R-CNN	Yolov3
0	Rec	63.41 ± 6.43	**85.23** ± **5.69**	78.02 ± 10.43
	Prec	87.84 ± 5.44	92.87 ± 2.87	**98.60** ± **0.57**
	MOTA	53.79 ± 8.71	**78.04** ± **7.15**	76.81 ± 10.71
	F1	73.53 ± 5.64	**88.82** ± **4.03**	86.80 ± 6.79
25	Rec	60.25 ± 4.52	**83.35** ± **4.95**	79.25 ± 9.70
	Prec	89.89 ± 4.33	92.08 ± 3.02	**98.06** ± **1.12**
	MOTA	52.82 ± 5.31	75.87 ± **5.89**	**77.65** ± 10.06
	F1	72.04 ± 3.59	**87.42** ± **3.34**	87.40 ± 6.38
50	Rec	60.96 ± 5.35	**86.27** ± **5.24**	79.18 ± 8.87
	Prec	90.84 ± 4.04	94.92 ± 1.34	**97.36** ± **0.67**
	MOTA	54.16 ± 6.14	**81.38** ± **5.06**	76.93 ± 8.56
	F1	72.84 ± 4.27	**90.30** ± **3.05**	87.09 ± 5.55

**Table 4:** Tracking results: D-SORT (all values in percentages, means over all video sequences, ± indicate the standard deviation σ).

% Expansion	Metrics	EspiNetV2	Faster R-CNN	Yolov3
0	Rec	74.21 ± 6.11	**87.97** ± **5.55**	83.22 ± 9.04
	Prec	78.00 ± 8.75	88.33 ± 3.52	**97.06** ± **1.18**
	MOTA	51.92 ± 12.86	75.71 ± **7.88**	**80.34** ± 9.81
	F1	75.87 ± 6.10	88.10 ± **4.14**	**89.42** ± 5.76
25	Rec	69.34 ± 4.98	83.48 ± **5.45**	**83.50** ± 8.41
	Prec	80.74 ± 6.60	86.87 ± 4.01	**96.37** ± **1.87**
	MOTA	51.83 ± 8.04	70.55 ± **7.81**	**80.54** ± 9.60
	F1	74.44 ± **4.12**	85.07 ± 4.15	**89.57** ± 5.66
50	Rec	70.84 ± 5.38	**88.15** ± **5.33**	83.49 ± 8.11
	Prec	82.58 ± 5.98	91.73 ± 2.01	**96.09** ± **0.59**
	MOTA	55.00 ± 7.75	**79.94** ± **5.44**	79.77 ± 8.01
	F1	76.10 ± 4.14	**89.83** ± **3.13**	89.16 ± 4.93

**Table 5:** Counting detection definitions (positive/negative).

	There Is a Crossing	There Is No Crossing
Crossing detected	TP	FP
Crossing not detected	FN	TN

**Table 6:** Counting results for 322 video sequences (all values in percentages, means over all video sequences, ± indicate the standard deviation σ).

Direction	Precision	Recall	F1	Accuracy
Alighting	95.49 ± 0.13	95.04 ± 0.12	95.10 ± 0.12	92.01 ± 0.12
Boarding	93.59 ± 0.05	98.82 ± 0.02	96.01 ± 0.03	92.47 ± 0.05
